# Clinical efficacy and safety of tripterygium wilfordii glycosides in the treatment of idiopathic membranous nephropathy: a systematic review and meta-analysis

**DOI:** 10.3389/fphar.2025.1652789

**Published:** 2025-10-31

**Authors:** Ying Liu, Keda Lu

**Affiliations:** ^1^ Zhejiang Chinese Medical University, Hangzhou, China; ^2^ Zhejiang Chinese Medical University Affiliated Third Hospital, Hangzhou, China

**Keywords:** tripterygium wilfordii glycosides, idiopathic membranous nephropathy, meta-analysis, systematic review, clinical efficacy, safety

## Abstract

**Background:**

Idiopathic membranous nephropathy (IMN) is a common cause of nephrotic syndrome in adults, with current immunosuppressive therapies often limited by incomplete efficacy, significant toxicity, and high cost. Extracts from Tripterygium wilfordii, particularly its glycosides (TWG), have emerged as a potential alternative with immunomodulatory properties.

**Objectives:**

To evaluate the clinical efficacy and safety of TWG in the treatment of IMN.

**Methods:**

We systematically searched PubMed, Embase, Cochrane Library, and Chinese databases from inception to September 2025. Randomized controlled trials (RCTs) and observational studies comparing TWG with standard therapies were included. Risk ratios (RR) and standardized mean differences (SMD) were pooled using a random-effects model.

**Results:**

This meta-analysis incorporated 20 studies (1,789 patients). TWG significantly improved the total response rate (RR = 1.27; 95% confidence interval (CI): 1.12–1.44), complete remission rate (RR = 1.81; 95% CI: 1.13–2.90), and reduced 24-h urinary protein (SMD = −2.09; 95% CI: 3.46 to −0.71) and recurrence risk (RR = 0.56; 95% CI: 0.37–0.86). However, the evidence was characterized by high heterogeneity (I^2^ > 50% for most efficacy outcomes) and a high risk of bias in 17 of the 20 included studies. No significant difference was observed in serum albumin (SMD = 1.20; 95% CI: 0.25–2.64) or the overall incidence of inadequately reported adverse events (RR = 0.93; 95% CI: 0.65–1.34).

**Conclusion:**

TWG may represent a beneficial therapeutic strategy for IMN, potentially improving remission rates and reducing proteinuria. However, the conclusiveness of these findings is constrained by the high risk of bias in the primary studies, substantial heterogeneity, and inadequate safety reporting. Future robust, multi-regional RCTs are required to definitively establish its efficacy and safety profile.

## 1 Introduction

Membranous Nephropathy (MN) is an autoimmune disease characterized by the deposition of immune complexes on the subepithelial side of the glomerular basement membrane. It represents one of the most common pathological types of nephrotic syndrome in adults, predominantly affecting middle-aged and elderly individuals with a mean age of onset between 50 and 60 years, and a significantly higher incidence in males compared to females. Epidemiological studies indicate that approximately 30%–40% of patients with Idiopathic Membranous Nephropathy (IMN) may experience spontaneous remission. However, about one-third of patients will progress to End-Stage Renal Disease (ESRD), underscoring the urgent need for effective interventions (Caravaca-Fontán et al., 2024).

The pathogenesis of MN long remained elusive until the proposal and refinement of the “podocyte antigen theory” brought about groundbreaking advances. This theory posits that IMN is essentially a kidney-specific autoimmune disease mediated by autoantibodies directed against specific antigens on the podocyte surface. A landmark discovery in 2009 identified the M-type phospholipase A2 receptor (PLA2R) as the primary target antigen in IMN, with anti-PLA2R antibodies detectable in approximately 70%–80% of patients (Beck et al., 2009). Subsequently, thrombospondin type-1 domain-containing 7A (THSD7A) was identified as a second major antigen in 2014, albeit with a lower prevalence (approximately 1%–5%), and some cases being associated with underlying malignancies (Tomas et al., 2014). In recent years, advancements in research technologies have led to the identification of a series of novel antigens, including Exostosin 1/2 (EXT1/2), Neural Epidermal Growth Factor-Like 1 (NELL-1), and Semaphorin 3B (SEMA3B), revealing that MN constitutes a group of disorders driven by different antibodies, yet sharing a common histopathological phenotype (Sethi and Fervenza, 2025). The autoimmune basis of IMN shares conceptual similarities with other chronic inflammatory diseases. For instance, in Rheumatoid Arthritis (RA), oxidative stress-driven protein glycation and glycoxidation lead to the formation of Advanced Glycation End products (AGEs), which act as neoantigens and amplify autoimmunity. These AGE-modified proteins activate macrophages via the Receptor for Advanced Glycation End products (RAGE) signaling pathway and promote the release of pro-inflammatory cytokines such as Interleukin-1 (IL-1), IL-6, and Tumor Necrosis Factor-α (TNF-α), thereby accelerating joint damage and systemic inflammation (Islam et al., 2018). This mechanism shares a fundamental commonality with IMN—the core concept of “autoantigens triggering aberrant immune responses” —wherein the production of autoantibodies against specific podocyte antigens ultimately leads to kidney injury.

Regarding clinical diagnosis, while renal biopsy remains the traditional gold standard for diagnosing MN, the procedure itself carries inherent risks due to its invasive nature. Given the high specificity and sensitivity of anti-PLA2R antibodies for IMN, international consensus now recommends a serology-based approach for non-invasive diagnosis in patients presenting with nephrotic syndrome, normal renal function, and after exclusion of secondary causes (De Vriese et al., 2016; Bobart et al., 2019). This strategy has been incorporated into international guidelines, such as those from KDIGO (Kidney Disease: Improving Global Outcomes), and has become part of standard clinical practice (Rovin et al., 2021). However, renal biopsy remains indispensable for patients with atypical clinical presentations, impaired renal function, or suspected secondary causes.

In terms of treatment strategy, the KDIGO guidelines recommend risk stratification based on parameters such as proteinuria levels, renal function, and antibody titers to guide individualized treatment plans (Rovin et al., 2021). All patients with IMN should receive foundational supportive care, including Angiotensin System Blockers (RASi) and Sodium-Glucose Cotransporter-2 Inhibitors (SGLT2i). For patients at moderate to high risk, immunosuppressive therapy is required in addition to supportive care. Commonly used first-line regimens include Rituximab (RTX), cyclophosphamide (CTX)-based protocols, or Calcineurin Inhibitors (CNI) (Rovin et al., 2021). However, each of these agents has its own limitations: CTX is associated with myelosuppression, gonadal toxicity, and long-term malignancy risk (Miller and Caza, 2023); CNIs have a high relapse rate upon discontinuation and potential nephrotoxicity with long-term use (Fervenza et al., 2019); while RTX is generally well-tolerated, concerns remain regarding infection risk, suboptimal response in some patients, and its relatively high cost (Scolari et al., 2021). Consequently, there is a persistent clinical need to identify alternative therapeutic agents with comparable efficacy, improved safety profiles, or better cost-effectiveness.

In summary, the limitations associated with current standard immunosuppressive regimens highlight the pressing need for novel treatment options. In this context, Tripterygium wilfordii glycosides (TWG), active components derived from the traditional Chinese herb Tripterygium wilfordii, have emerged as a promising potential alternative therapy in the management of IMN, owing to their unique immunomodulatory, anti-inflammatory, and direct podocyte-protective properties (Zhang et al., 2023).

TWG is a mixture of glycosides extracted from the root of Tripterygium wilfordii. Its core active ingredient, triptolide, has been demonstrated to possess potent immunosuppressive and anti-inflammatory activities. Numerous clinical studies have shown that TWG can not only be used as monotherapy for low-to-moderate risk IMN patients, effectively reducing proteinuria and inducing clinical remission (Qiu et al., 2021), but its efficacy and complete remission rates are significantly superior when combined with low-dose corticosteroids, demonstrating a synergistic effect (Ai and Wang, 2022). Furthermore, observational data suggest that the relapse rate after achieving remission with TWG treatment is relatively low, constituting a potential advantage over some CNIs (Zhao, 2022).

Despite these promising findings, the definitive role of TWG in IMN treatment remains controversial. This uncertainty stems primarily from the fragmentation of existing evidence: firstly, conclusions regarding the efficacy of TWG across various studies are inconsistent, particularly in head-to-head comparisons with different active drugs; secondly, and more critically, reporting of TWG’s well-documented potential toxicities (such as hepatotoxicity, reproductive toxicity, and gastrointestinal adverse effects) (Xu et al., 2019; Wei et al., 2019) is often incomplete and non-systematic in primary studies, hindering a comprehensive risk-benefit assessment. Moreover, although related systematic reviews exist, they are often limited by the small number of included studies, failure to incorporate the most recent clinical evidence, or lack of distinction and appropriate handling of different study designs (e.g., randomized controlled trials vs. observational studies), thereby limiting the reliability and generalizability of their conclusions.

Therefore, there is a compelling need for a methodologically rigorous, up-to-date, and comprehensive systematic review and meta-analysis to synthesize all available evidence, to objectively quantify the efficacy and safety of TWG in treating IMN. To overcome the limitations of previous reviews, this study will systematically search both English and Chinese databases, include randomized controlled trials and observational studies, and differentiate and combine evidence from different design types accordingly. While evaluating efficacy outcomes (such as overall response rate, complete remission rate, and reduction in proteinuria), particular emphasis will be placed on the systematic summarization and analysis of adverse reactions, aiming to provide a more reliable evidence base to inform its clinical application.

## 2 Materials and methods

### 2.1 Protocol and reporting standards

This systematic review and meta-analysis was conducted in accordance with the referred Preferred Reporting Items for Systematic Reviews and Meta-Analyses (PRISMA) 2020 statement. The study protocol was registered in PROSPERO (Registration number: CRD420251146378).

### 2.2 Information sources and search strategy

A comprehensive literature search was conducted to identify all relevant randomized controlled trials (RCTs) and observational studies that investigated the efficacy and/or safety of Tripterygium wilfordii preparations (e.g., Tripterygium glycosides, multi-glycoside of Tripterygium wilfordii) in patients with idiopathic membranous nephropathy (IMN). The search encompassed the following electronic databases from their inception until 11 September 2025:

English Databases: PubMed (via NCBI), Embase (via Ovid), Cochrane Central Register of Controlled Trials (CENTRAL), and Web of Science Core Collection.

Chinese Databases: China National Knowledge Infrastructure (CNKI), WanFang Data, and VIP Database.

The search strategy was designed around key concepts related to the intervention (Tripterygium wilfordii and its preparations) and the disease (idiopathic membranous nephropathy). Search terms included both controlled vocabulary (e.g., MeSH terms in PubMed, Emtree terms in Embase) and free-text words (e.g., “tripterygium wilfordii”, “tripterygium glycosides”, “TWG”, “idiopathic membranous nephropathy”, “membranous nephropathy”, “IMN”). The specific search strategies for each database were tailored to its structure and query language, and are provided in full in Supplementary Table S1.

### 2.3 Eligibility criteria

The inclusion criteria were formulated according to the PICOS framework. Studies were included if they met all the following criteria: (1) Population: Adult patients (≥18 years) diagnosed with IMN, confirmed either by renal biopsy or serologically based on a positive anti-PLA2R antibody test, in accordance with international guidelines (e.g., KDIGO). (2) Intervention: Treatment with Tripterygium wilfordii glycosides (TWG), either as monotherapy or in combination with other treatments. (3) Comparator: Conventional therapies, including corticosteroids, cyclophosphamide, tacrolimus, or supportive care. (4) Outcomes: Reporting of at least one of the following predefined outcomes: total clinical response rate (complete remission + partial remission), complete remission, partial remission, urinary protein excretion, serum albumin level, recurrence rate, or adverse events. Complete remission was defined as urinary protein <0.3 g/d with normal serum albumin and serum creatinine levels (in accordance with KDIGO guidelines). Partial remission was defined as a >50% reduction in urinary protein from baseline, with a final urinary protein level between 0.3 and 3.5 g/d. Relapse was defined as an increase in urinary protein to >3.5 g/d or a >50% increase from the lowest level after achieving complete or partial remission. (5) Study Design: Randomized controlled trials (RCTs), cohort studies, or other controlled studies published in Chinese or English. The potential for language bias is acknowledged as a limitation. In cases of missing or insufficient data, corresponding authors were contacted via email to obtain necessary information. Studies were excluded if authors did not respond. For multiple publications stemming from the same trial, only the report with the most comprehensive data was included.

### 2.4 Exclusion criteria

(1) Non-clinical or non-comparative study designs, including animal experiments, *in vitro* studies, case reports, case series with fewer than 10 participants, reviews, editorials, and conference abstracts. (2) Studies involving populations with secondary membranous nephropathy or those diagnosed with nephrotic syndrome without confirmed IMN. (3) The intervention includes studies where the glycosides from Tripterygium wilfordii have not been clearly defined and isolated. (4) Studies lacking a control group receiving conventional therapy or supportive care; those failing to report any prespecified efficacy or safety outcomes; or duplicate publications/secondary analyses of already included patient cohorts. (5) Significant imbalance in baseline characteristics between the TWG and control groups, indicating non-comparability.

### 2.5 Data extraction

The following data were extracted from the included studies using a pre-designed data extraction form:

Study identification: first author, publication year, and country. Study characteristics: design (e.g., RCT, prospective cohort), sample size, and follow-up duration. Patient demographics: age range and gender distribution. Clinical details: specific diagnostic criteria for IMN and baseline levels of key indicators (e.g., urinary protein, serum albumin). Intervention details: TWG dosage and treatment regimen, as well as the specific protocols used in the comparator groups (e.g., drug, dosage). Outcome data: results pertaining to total clinical response, complete and partial remission, 24-h urinary protein, serum albumin, recurrence rate, and adverse events. Results of the risk of bias or quality assessments.

The data extraction was performed independently by two reviewers (Ying Liu and Yuan Zhuang).

### 2.6 Risk of bias and quality assessment

The quality and risk of bias of the included studies were assessed using standardized tools specific to their design. For randomized controlled trials (RCTs), the Cochrane Risk of Bias tool (RoB 2) was employed. Based on the domain-level judgments, each RCT was assigned an overall risk of bias classification of “low risk,” “some concerns,” or “high risk.”For observational studies (including prospective and retrospective cohorts), the Newcastle-Ottawa Scale (NOS) was used. The NOS assesses studies on three domains: the selection of the study groups, the comparability of the groups, and the ascertainment of the outcome of interest. Based on their total scores, observational studies were categorized as high-quality (score of 7–9), medium-quality (score of 4–6), or low-quality (score of 0–3).

Two reviewers (Ying Liu and Yuan Zhuang) independently conducted all assessments. Any discrepancies were resolved through discussion or, when necessary, by consulting a third senior researcher (KeDa Lu) for a final decision.

### 2.7 Statistical analysis

All meta-analyses were performed using Stata software, version 14.2 (StataCorp, College Station, TX, United States of America). A random-effects inverse-variance model was employed for all pooled analyses to account for the anticipated clinical and methodological heterogeneity among the included studies. Treatment effects for dichotomous outcomes were expressed as risk ratios (RR) with 95% confidence intervals (CIs), while continuous outcomes were expressed as standardized mean differences (SMD) with 95% CIs. The Mantel-Haenszel method was used to pool RRs, and the inverse-variance method was used for SMDs.

Statistical heterogeneity was assessed using the Cochran’s Q test (with a significance level of p < 0.10) and quantified using the I^2^ statistic. An I^2^ value greater than 50% was considered to indicate substantial heterogeneity. To explore potential sources of heterogeneity for the primary outcome (total clinical response), pre-specified subgroup analyses were conducted based on the following study-level characteristics: risk of bias (high vs. low/some concerns), study design (randomized vs. observational), comparator type (immunosuppressive agents vs. supportive therapies), TWG administration strategy (monotherapy vs. combination therapy), follow-up duration, and sample size. Subgroup differences were formally tested using a mixed-effects model, with the Hartung-Knapp-Sidik-Jonkman (HKSJ) adjustment applied to the random-effects model to enhance the robustness of intervals, particularly given the inclusion of some smaller studies. The statistical significance of subgroup differences was assessed using the Q test for subgroup differences, with an interaction p-value <0.05 considered statistically significant.

Publication bias was assessed visually using funnel plots and statistically using Egger’s regression test, but only for outcome measures that included 10 or more studies, as the power of these tests is insufficient with a smaller number of studies. A two-sided p-value <0.05 was considered statistically significant for the overall effect size.

### 2.8 GRADE assessment

The certainty of evidence for each primary outcome was assessed using the Grading of Recommendations Assessment, Development and Evaluation (GRADE) framework. We evaluated the following domains for each outcome: risk of bias, inconsistency, indirectness, imprecision, and publication bias. The overall certainty of evidence was categorized as high, moderate, low, or very low. Two reviewers (Ying Liu and Yuan Zhuang) independently performed the GRADE assessments. Any discrepancies were resolved through discussion or, when necessary, by consulting a third senior researcher (Keda Lu). The results of this assessment are presented in a ‘Summary of Findings’ table in the Results section (Table 1).

**TABLE 1:** Summary of findings (GRADE): Tripterygium wilfordii glycosides (TWG) versus control for idiopathic membranous nephropathy (IMN).

Outcomes	Relative effect (95% CI)	Quantity of participants (studies)	Certainty of the evidence (GRADE)	Comments
Total clinical response	RR 1.27 (1.14–1.41)	1,789 (19)	@@○○ LOW a, b	TWG likely increases the total response rate
Complete remission	RR 1.81 (1.13–2.90)	602 (7)	@@○○ LOW a, c	TWG may increase the complete remission rate
Partial remission	RR 0.99 (0.80–1.24)	602 (6)	@@○○ LOW a, d	TWG may result in little to no difference in partial remission
24-h urinary protein	SMD -2.09 (−3.46 to −0.71)	442 (5)	@○○○ VERY LOW a, e	TWG may reduce urinary protein, but the evidence is very uncertain
Recurrence rate	RR 0.56 (0.37–0.86)	251 (3)	@@○○ LOW f	TWG may reduce the risk of recurrence
Overall incidence of adverse events	RR 0.93 (0.65–1.34)	854 (9)	@@○○ LOW a, g	The evidence is very uncertain about the effect of TWG on overall adverse events

Abbreviations: CI, confidence interval; RR, risk ratio; SMD, standardised mean difference.

### 2.9 Ethical compliance

The meta-analysis was limited to published studies that explicitly reported ethical approval from pertinent institutional review boards or ethics committees. Individual patient data (IPD) were not utilized. Studies without explicit ethical information were considered in sensitivity analyses and discussed as a limitation. Compliance with the Declaration of Helsinki was assumed for all included studies.

## 3 Results

### 3.1 Study selection

**FIGURE 1:**
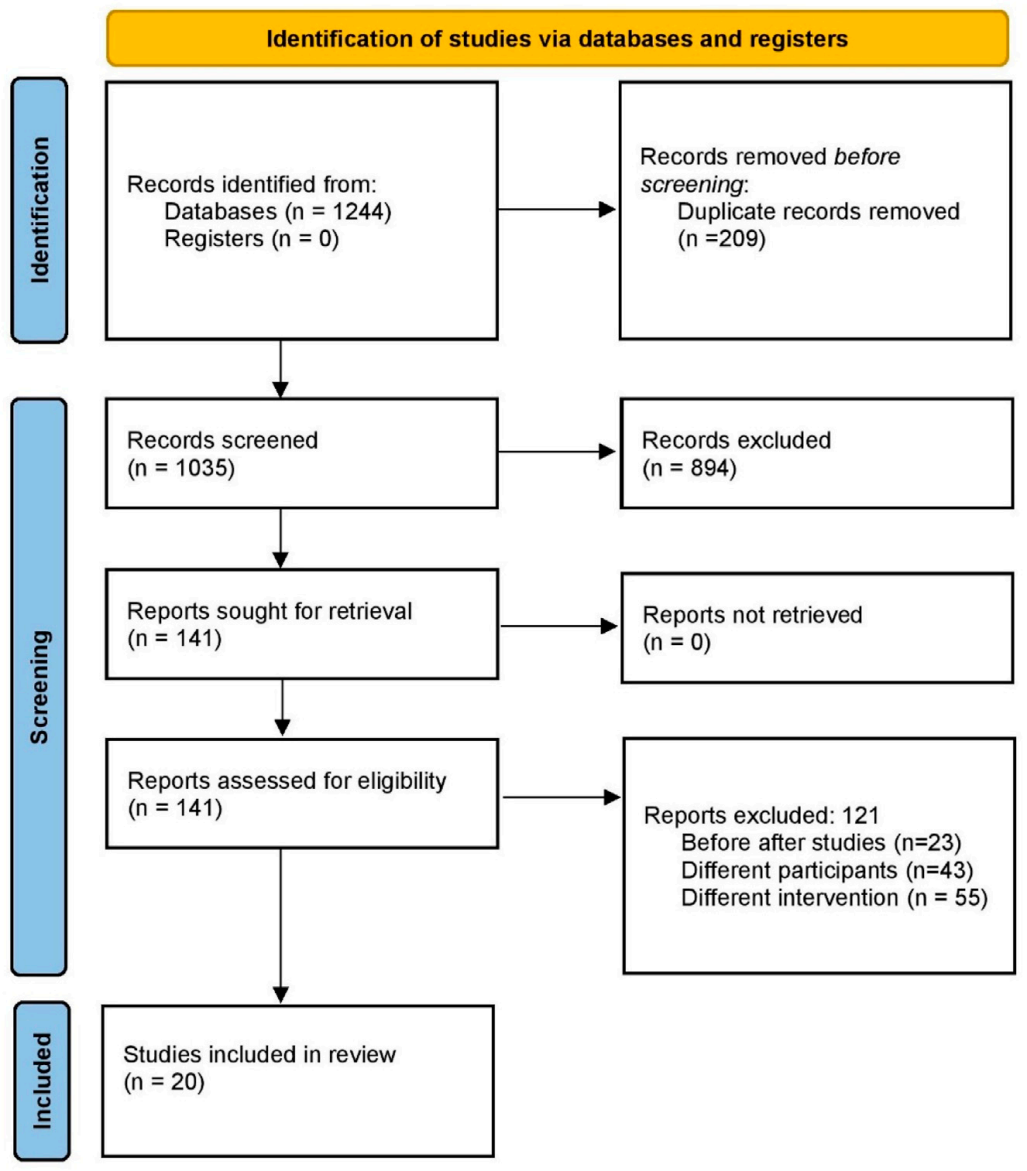
PRISMA flow diagram of study selection.

The database search initially identified 1,244 unique records. After removing duplicates and screening titles and abstracts, 141 articles were selected for full-text review. A total of 20 (Stanescu et al., 2011; Shao et al., 2021; Wu et al., 2022; Li et al., 2022; Page et al., 2021; Guo et al., 2021; Liu et al., 2015; Shang et al., 2018; Zuo et al., 2014; Zhou et al., 2020; Yang and Xie, 2018; Yang et al., 2015; Liu et al., 2017; Ling et al., 2015; Di, 2019; Qu et al., 2016; Jiang, 2023; Liang et al., 2020; Peng et al., 2015; Gao et al., 2017) studies met the eligibility criteria and were included in the meta-analysis, encompassing 1,789 patients with IMN. The study selection process is illustrated in the PRISMA flow diagram (Figure 1).

Flowchart summarizing the systematic screening process of identified records through database searching, title/abstract screening, full-text assessment, and final inclusion in qualitative and quantitative synthesis (n = 20 studies).

### 3.2 Characteristics of included studies

Among the 20 studies, 17 were RCTs, 2 were prospective cohort studies, and 1 was retrospective. All were conducted in China between 2009 and 2023. Sample sizes ranged from 30 to 240. TWG were used either as monotherapy or in combination with immunosuppressive agents. Control groups included steroids, cyclophosphamide, tacrolimus, or angiotensin receptor blockers. Follow-up durations ranged from 3 to 12 months. A high risk of bias was identified in seventeen studies. Table 2 provides a comprehensive overview of the study characteristics.

**TABLE 2:** Characteristics of included studies.

Author and year	Study design	Country	Sample size	Groups and gender	Mean age (in years	Diagnostic method	Disease and classification	Intervention details	Comparator details	Follow-up duration	Outcome definitions applied	Risk of bias
Jin 2019	RCT	China	63	Total (M: 33, F: 30)	NR	Biopsy-proven	IMN (all stages – stage II commonest)	Tripterygium Wilfordii Glycosides 1 mg/kg/day three times a day	Benazepril Hydrochloride tablets	12 weeks	Other	High
Diao 2017	RCT	China	100	OG: (M:29, F:31) CG: (M:32, F:18)	43.96	Biopsy-proven	IMN (Stages NR)	Tripterygium Wilfordii with YiqiHuoxueLishui formulation	Prednisolone tablets	6 months	Other	High
Feng 2015	RCT	China	240	CG: (M:76, F:44 OG: (M:74, F:46)	45.7 ± 10.3	Biopsy-proven	IMN (Stages 1–3)	Tripterygium Wilfordii 20 mg orally three times per day	Small dose of hormone	6 months	Other	Low
Gao 2013	RCT	China	30	Total (M:19, F:11)	NR	Biopsy-proven	IMN (Stages 1–3)	Tripterygium Wilfordii + Control group drug	Tacrolimus + corticosteroids	6 months	Other	High
Gao 2017	RCT	China	52	OG: (M:16, F:10) CG: (M:15, F:11)	NR	Biopsy-proven	IMN (Stages NR)	Tripterygium Wilfordii + low dose hormone therapy	Antiplatelet therapy	12 months	Other	High
Guo 2021	Retrospective	China	55	OG: (M:20, F:15) CG: (M:11, F:9	NR	Biopsy-proven	IMN (Stages NR)	Tripterygium Wilfordii + Angiotensin receptor blockers	Angiotensin receptor blockers	9 months	KDIGO-like	High
Jiang 2023	RCT	China	58	OG: (M:16, F:13) CG: (M:17, F:12)	NR	Biopsy-proven	IMN (Stages NR)	Tripterygium Wilfordii + low dose hormone therapy	Cyclophosphamide	6 months	Other	High
Liang 2020	RCT	China	92	OG: (M:29, F:17) CG: (M:25, F:21)	NR	Biopsy-proven	IMN (Stages NR)	Tripterygium Wilfordii	Tacrolimus	12 months	Other	High
Ling 2015	RCT	China	85	OG: (M:20, F:22) CG: (M:21, F:22)	NR	Biopsy-proven	IMN (Stages NR)	Tripterygium Wilfordii 40 mg three times per day for 12 months	Oral valsartan	12 months	KDIGO-like	High
Liu 2009	RCT	China	84	OG: (M:31, F:12) CG: (M:30, F:11)	NR	Biopsy-proven	Biopsy-proven IMN with nephrotic syndrome	Tripterygium wilfordii: 120 mg/d for 3 months	Steroids	12 months	Other	High
Liu 2015	Prospective cohort study	China	53	OG: (M:12, F:11) CG: (M:22, F:8)	NR	Biopsy-proven	Biopsy-proven IMN of all stages	Tripterygium wilfordii + prednisone	Tacrolimus + prednisone	9 months	KDIGO-like	High
Liu 2017	RCT	China	84	OG: (M:27, F:13) CG: (M:28, F:16)	NR	Biopsy-proven	IMN (Stages 1–2)	Tripterygium wilfordii	Cyclosporine	6 months	KDIGO-like	High
Peng 2015	RCT	China	40	OG: (M:8, F:12) CG: (M:9, F:11)	NR	Biopsy-proven	IMN (Stages NR)	Tripterygium wilfordii	Tacrolimus	6 months	KDIGO-like	Low
Qu 2016	RCT	China	56	OG: (M:9, F:19) CG: (M:11, F:17)	NR	Biopsy-proven	IMN (Stages NR)	Tripterygium wilfordii 20 mg three times per day	Oral losartan potassium	12 months	Other	High
Shang 2018	Retrospective	China	120	OG: (M:18, F:27) CG: (M:37, F:38)	NR	Biopsy-proven	Biopsy-proven IMN of all stages	Tripterygium wilfordii + Tacrolimus	Tacrolimus	16 months	KDIGO-like	High
Xie 2018	RCT	China	180	OG: (M:48, F:42) CG: (M:46, F:44)	NR	Biopsy-proven or Clinical	IMN (Stages NR)	Tripterygium Wilfordii with YiqiHuoxueLishui formulation	Prednisone + cyclophosphamide	6 months	Other	Low
Yang 2015	RCT	China	90	OG: (M:25,F: 20) CG: (M:28, F:17	NR	Biopsy-proven	IMN (Stages NR)	Tripterygium Wilfordii	Mycophenolate mofetil	NR	KDIGO-like	High
Yang 2018	RCT	China	91	OG: (M:27, F:20) CG: (M:25, F:19	NR	Biopsy-proven	IMN (Stages 1–2)	Tripterygium Wilfordii	Benner Pury Treatment	NR	Other	High
Zhou 2020	RCT	China	76	OG: (M:21, F:17) CG: (M:28, F:10)	NR	Biopsy-proven	IMN (Stages NR)	Tripterygium Wilfordii + Prednisone + cyclophosphamide + Tacrolimus	Prednisone + cyclophosphamide	3 months	KDIGO-like	High
Zuo 2014	RCT	China	100	OG: (M:31, F:19 CG: (M:34, F:16)	NR	Biopsy-proven	IMN (Stages NR)	Tripterygium Wilfordii	Tacrolimus	12 months	Other	High

RCT, randomized controlled trial; IMN, idiopathic membranous nephropathy; NR, not reported; OG, observation group; CG, control group; M, male, F, female.

NR = Not Reported by original article. Several studies did not report mean age (e.g., Di 2019, Feng 2015, Gao 2013) or detailed disease staging. These missing data were acknowledged in the risk of bias assessments and discussed as a limitation in the main text.

### 3.3 GRADE assessment of evidence

The certainty of evidence for the key outcomes, as assessed by the GRADE methodology, is summarized in Table 3. The evidence was rated as low or very low for all outcomes, primarily due to the high risk of bias in the included studies, substantial heterogeneity, and imprecision in the effect estimates.

**TABLE 3:** Subgroup analysis of the total response rate comparing TWG versus control treatments in idiopathic membranous nephropathy.

Subgroup	k	Pooled RR (95% CI)	τ^2^	I^2^	Q	p-value (within)	p-value (between)
Risk of Bias							0.1752
High	17	1.24 [1.08, 1.43]	0.0423	65.6%	46.55	<0.0001	
Low	2	1.46 [0.42, 5.11]	0.0119	61.2%	2.58	0.1083	
Study Design							0.0279
RCT	16	1.31 [1.18, 1.45]	0.0182	55.0%	33.31	<0.0001	
Retrospective	2	1.13 [0.81, 1.57]	0.0216	25.5%	10.59	0.005	
Prospective	1	0.97 [0.79, 1.19]	—	—	—	—	
Follow-up Duration							0.8126
<6 months	3	1.39 [0.76, 2.53]	0.0353	58.9%	4.86	0.088	
6 months	9	1.21 [0.95, 1.55]	0.0669	78.1%	36.52	<0.001	
>6 months	5	1.36 [0.93, 1.98]	0.0626	71.5%	14.06	0.015	
Not Reported (NA)	2	1.25 [0.79, 1.96]	0	0.0%	0.28	0.597	
Comparator Type							0.9051
Supportive	6	1.27 [1.08, 1.48]	∼0	24.2%	6.59	0.252	
Immunosuppressant	13	1.25 [1.05, 1.49]	0.0577	76.2%	50.47	<0.001	
TWG Use Type							0.6058
Monotherapy	10	1.31 [1.09, 1.57]	0.0396	72.0%	32.15	<0.001	
Combination	9	1.23 [0.98, 1.54]	0.0501	67.5%	24.60	<0.001	
Sample Size							0.8479
<100	13	1.26 [1.07, 1.49]	0.0411	62.8%	32.24	<0.001	
≥100	6	1.29 [0.98, 1.71]	0.0499	79.6%	24.54	<0.001	

GRADE Working Group grades of evidence.

High certainty: We are very confident that the true effect lies close to that of the estimate of the effect.

Moderate certainty: We are moderately confident in the effect estimate: The true effect is likely to be close to the estimate of the effect, but there is a possibility that it is substantially different.

Low certainty: Our confidence in the effect estimate is limited: The true effect may be substantially different from the estimate of the effect.

Very low certainty: We have very little confidence in the effect estimate: The true effect is likely to be substantially different from the estimate of effect.

Explanations.

a. Risk of bias: Downgraded by one level. The majority of included studies (17/20) were judged to have a high risk of bias.
b. Inconsistency: Downgraded by one level for substantial heterogeneity (I^2^ = 68.6%).
c. Imprecision: Downgraded by one level. The confidence interval is wide and includes both a clinically important benefit and a marginal benefit.
d. Imprecision: Downgraded by one level. The confidence interval includes both no effect and appreciable harm or benefit.
e. Inconsistency and Imprecision: Downgraded by two levels. One level for very high heterogeneity (I^2^ = 96.9%) and one level for serious imprecision due to a very wide confidence interval.
f. Imprecision: Downgraded by one level due to the small number of events and included studies.
g. Indirectness/Reporting bias: Downgraded by one level. Adverse events were inconsistently and likely incompletely reported, failing to adequately capture the well-documented specific toxicities of TWG.

### 3.4 Clinical response

**FIGURE 2:**
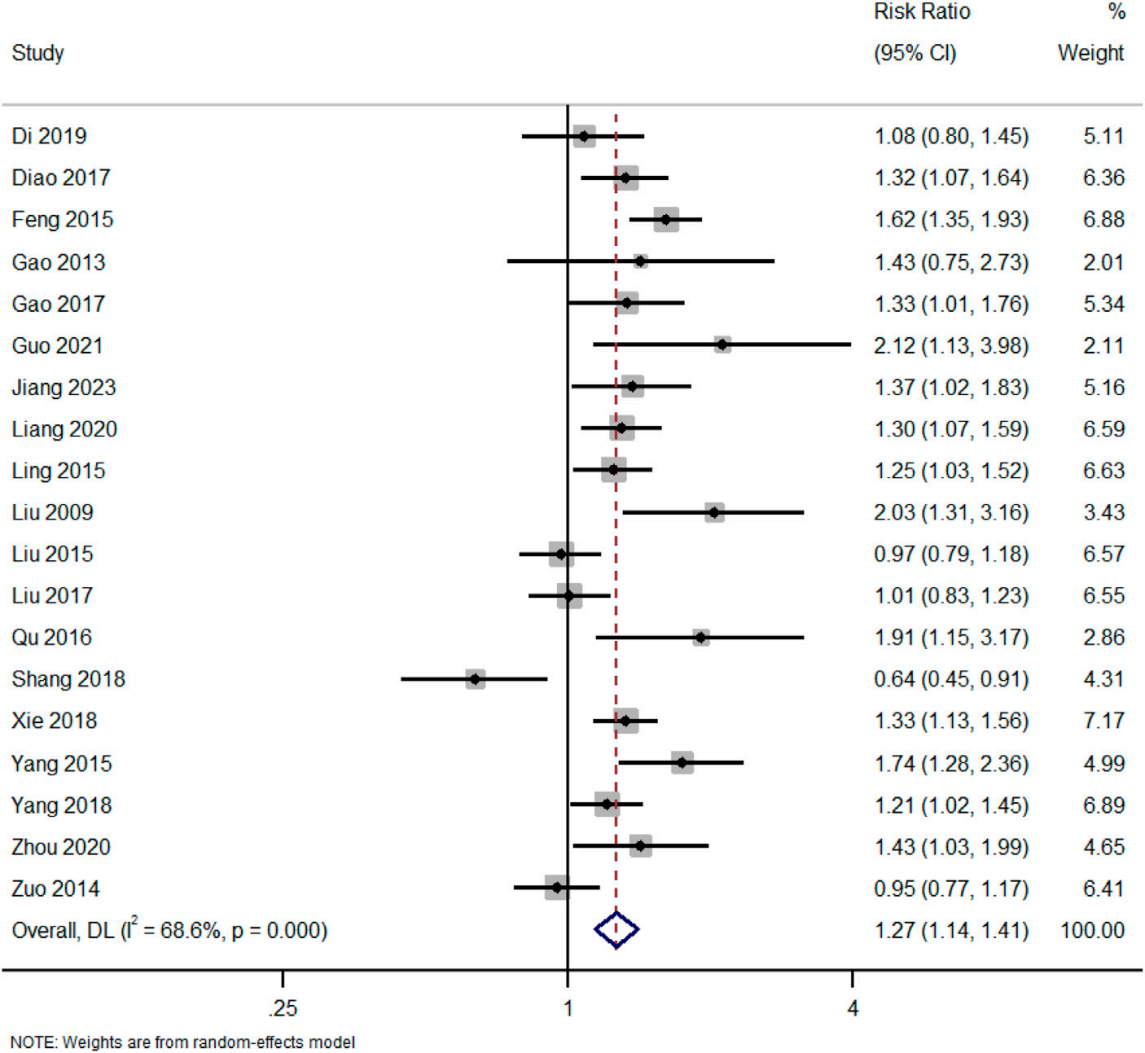
Forest plot of total clinical response (complete or partial remission).

In addition to total clinical response, separate analyses were conducted for complete and partial remission outcomes: A total of 19 studies reported data on total clinical response (complete or partial remission). Figure 2. Forest Plot of Total Clinical Response (Complete or Partial Remission). RR, risk ratio; CI, confidence interval.

Pooled RR comparing total clinical response rates between patients receiving TWG and control therapies across 19 studies. A significant overall benefit of TWG was observed (RR = 1.27; 95% CI: 1.14–1.41; I^2^ = 68.6%).

### 3.5 Complete and partial remission

In addition to total clinical response, separate analyses were conducted for complete and partial remission outcomes.

#### 3.5.1 Complete remission

**FIGURE 3:**
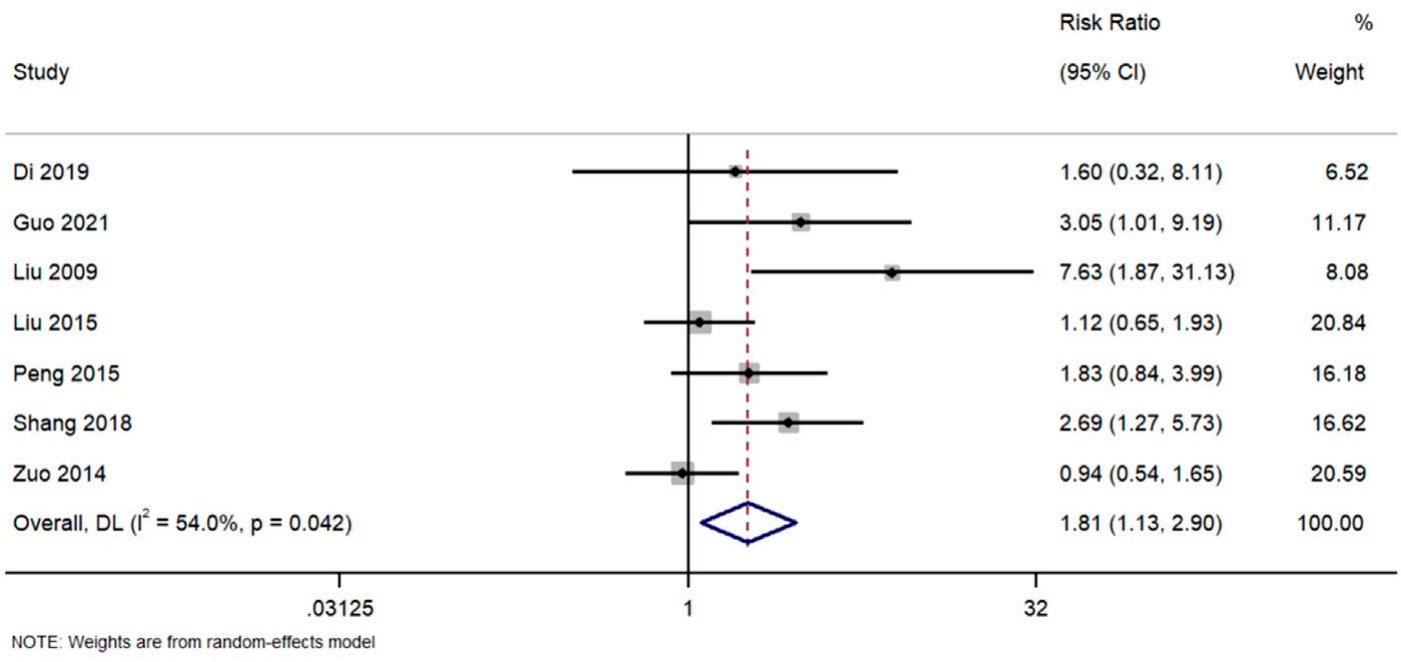
Forest plot of complete remission rates in TWG versus control groups.

Complete remission was observed in seven studies. Figure 3. Forest Plot of Complete Remission Rates in TWG versus Control Groups. RR, risk ratio; CI, confidence interval. Although most studies yielded positive outcomes, two (Zuo 2014, Liu 2015) reported RR close to 1.0, indicating heterogeneity in treatment response. Random-effects modeling was employed to address this variability. The moderate heterogeneity (I^2^ = 54%) suggests potential variation in study populations, treatment protocols, or outcome definitions.

Meta-analysis of seven studies assessing complete remission outcomes. TWG was associated with a significantly higher complete remission rate compared to controls (RR = 1.81; 95% CI:1.13–2.90; I^2^ = 54%).

#### 3.5.2 Partial remission

**FIGURE 4:**
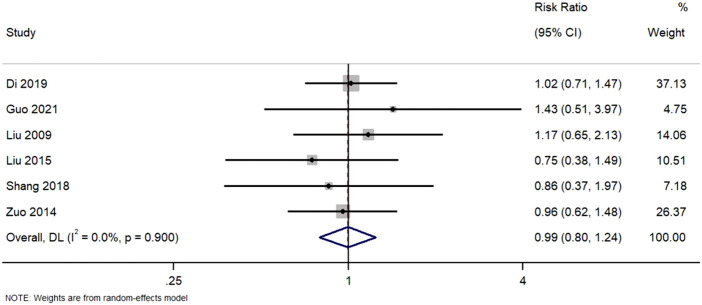
Forest plot of partial remission rates in TWG versus control groups.

Six studies reported partial remission. Figure 4. Forest Plot of Partial Remission Rates in TWG versus Control Groups. RR, risk ratio; CI, confidence interval. All studies demonstrated point estimates near unity, with confidence intervals encompassing 1.0. The homogeneity observed (I^2^ = 0%) suggests consistent findings across trials, supporting the lack of differential efficacy in achieving partial remission.

Analysis of six studies reporting partial remission. No statistically significant difference was observed between TWG and control therapies (RR = 0.99; 95% CI: 0.80–1.24; I^2^ = 0%).

### 3.6 Subgroup analyses

To investigate the origins of heterogeneity in the overall clinical response, subgroup analyses were performed (Table 3):

Risk of bias: TWG demonstrated consistent benefit across risk levels. High-risk studies (n = 17) showed RR = 1.24, and low-risk studies (n = 2) had RR = 1.46, though the latter was imprecise due to small sample size. No significant subgroup difference was detected (p = 0.175), suggesting the observed effect was not solely driven by bias.

Study design: The treatment effect was statistically stronger in RCTs (RR = 1.31) compared to observational studies, where results were more variable. The significant subgroup difference (p = 0.028) supports greater confidence in findings from randomized evidence.

Comparator type: TWG was effective against both supportive care (RR = 1.27) and immunosuppressants (RR = 1.25). The lack of subgroup difference (p = 0.91) suggests TWG’s benefit is broadly comparable across standard treatment types.

TWG use strategy: Effect sizes were similar whether TWG was used as monotherapy (RR = 1.31) or combined with conventional agents (RR = 1.23), with no statistically significant difference (p = 0.61). This implies flexibility in TWG treatment strategies.

Sample size: Studies with <100 participants showed RR = 1.26, while those with ≥100 showed RR = 1.29, with no significant subgroup difference (p = 0.85), indicating effect size stability across study sizes.

Follow-up duration: No meaningful subgroup differences were observed across <6, 6, or >6 months follow-up periods (p = 0.81), suggesting the clinical benefit of TWG is maintained across short-to moderate-term durations.

These findings indicate that the efficacy of TWG is generally consistent across methodological and clinical contexts, with stronger and more precise effects observed in randomized studies.

### 3.7 Biochemical parameters

#### 3.7.1 Serum albumin

**FIGURE 5:**
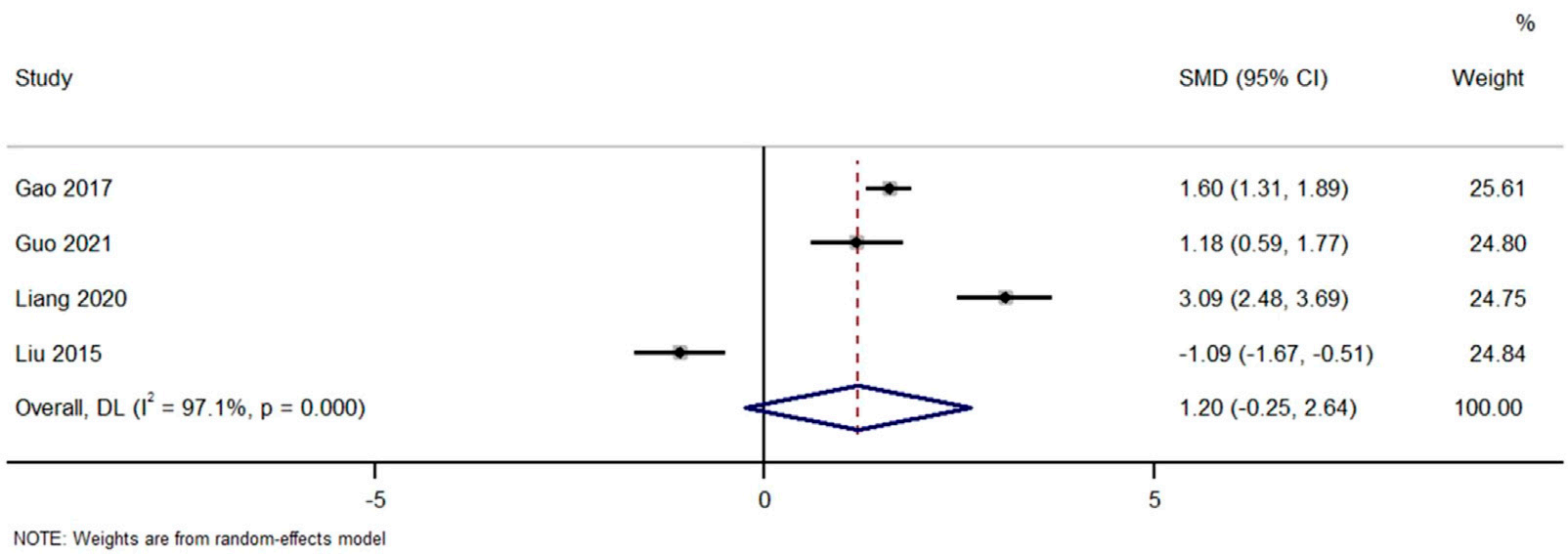
Forest plot of change in serum albumin levels with TWG versus controls.

Figure 5 Forest Plot of Change in Serum Albumin Levels with TWG versus Controls. SMD, standardized mean difference; CI, confidence interval.

Across studies comparing the effect of TWG on serum albumin. No significant difference was found (SMD = 1.20; 95% CI: 0.25 to 2.64; I^2^ = 97.1%), with high heterogeneity noted.

**FIGURE 6:**
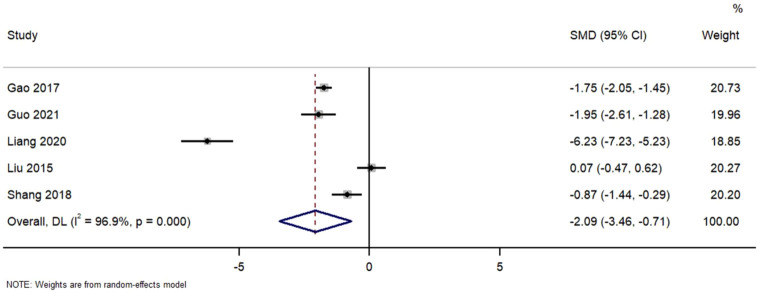
Forest plot of urinary protein reduction in TWG versus control groups.

Figure 6 Forest Plot of Urinary Protein Reduction in TWG versus Control Groups. SMD, standardized mean difference; CI, confidence interval.

Pooled analysis of urinary protein excretion outcomes. TWG treatment significantly reduced proteinuria compared to control (SMD = −2.09; 95% CI: 3.46 to −0.71; I^2^ = 96.9%)

### 3.8 Recurrence rate

**FIGURE 7:**
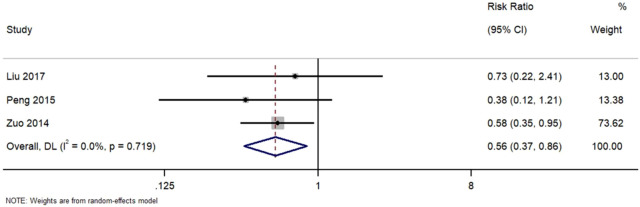
Forest plot of recurrence rate in TWG versus control groups.

Figure 7 Forest Plot of Recurrence Rate in TWG versus Control Groups. RR, risk ratio; CI, confidence interval.

Three studies reported recurrence outcomes. TWG treatment was associated with a significantly lower recurrence risk (RR = 0.56; 95% CI: 0.37–0.86; I^2^ = 0%).

### 3.9 Adverse events

**FIGURE 8:**
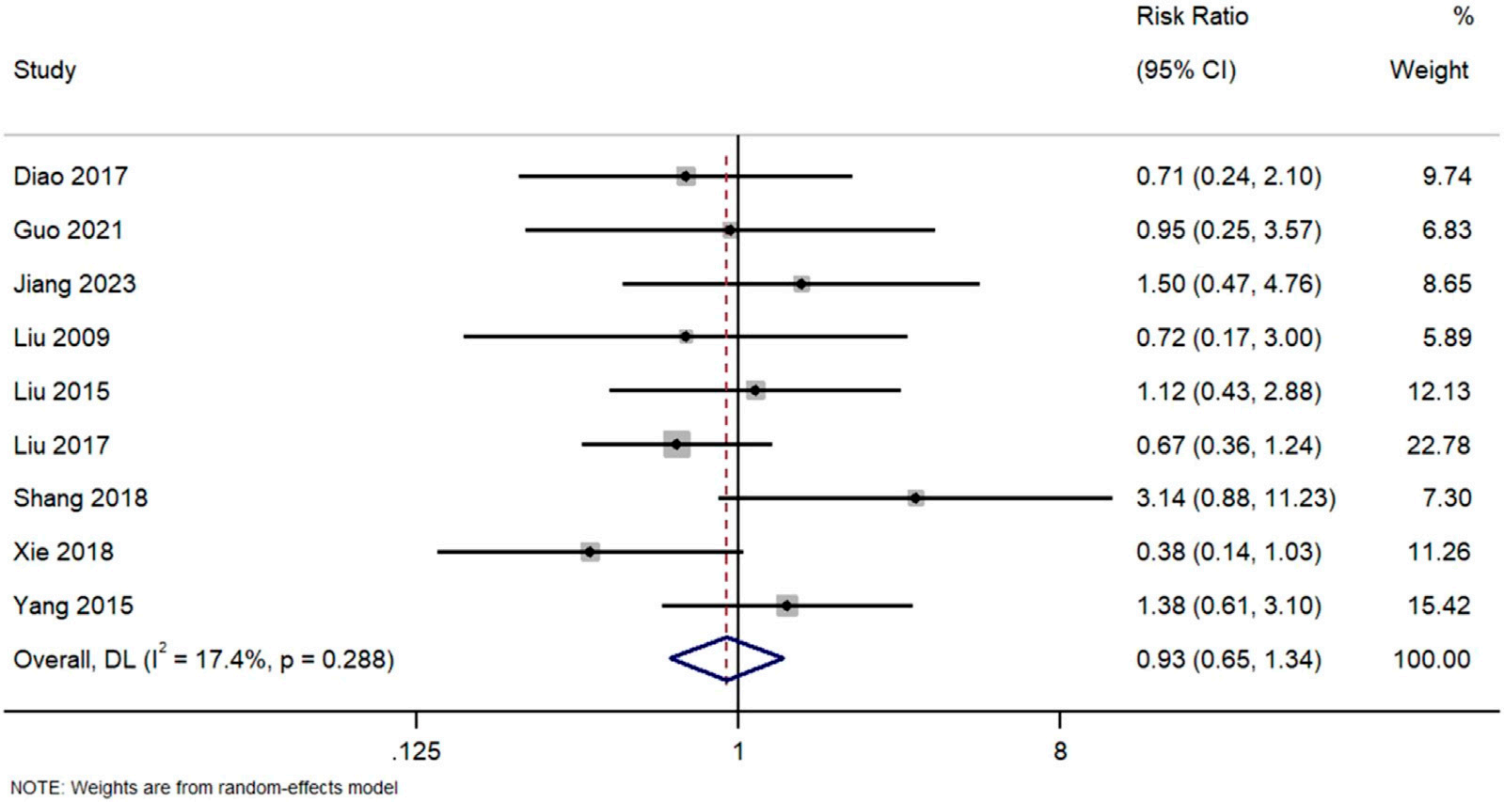
Sis of nine studies comparing adverse event rates.

Figure 8 Forest Plot of Overall Adverse Event Rates in TWG versus Control Groups. RR, risk ratio; CI, confidence interval. Indicating that the pooled analysis of reported events showed no statistically significant difference in overall adverse event rates between groups. However, this finding must be interpreted in the context of inconsistent and likely incomplete reporting across studies, which fails to adequately capture the well-documented, specific toxicities of TWG (e.g., hepatotoxicity, reproductive toxicity) (Figure 8).

No significant difference was observed bForest Plot of Overall Adverse Event Rates in TWG versus Control Groups. Meta-analysis between TWG and control groups (RR = 0.93; 95% CI: 0.65–1.34; I^2^ = 17.4%). However, in a subgroup of four studies that reported gastrointestinal side effects, TWG was associated with a non-significant trend toward higher GI toxicity (RR = 1.32; 95% CI: 0.98–1.78).

## 4 Discussion

The findings of this meta-analysis suggest potential clinical implications. The results demonstrate that, compared with control treatments, TWG therapy was significantly associated with an improved overall clinical response (risk ratio [RR] = 1.27; 95% confidence interval [CI]: 1.12–1.44; p < 0.001). Furthermore, TWG significantly increased the rate of complete remission (RR = 1.81; 95% CI: 1.13–2.90), while no significant difference was observed in partial remission rates between treatment groups (RR = 0.99; 95% CI: 0.80–1.24). This differential effect on remission types indicates that the therapeutic benefit of Tripterygium wilfordii glycosides (TWG) may be primarily driven by the induction of complete remission, which aligns with its postulated role as a disease-modifying agent in IMN. These findings are consistent with the known pharmacological profile of TWG, which includes immunomodulatory and podocyte-protective effects (Zhang et al., 2023). Collectively, these data position TWG as a potential therapeutic alternative for IMN patients who are intolerant or unresponsive to first-line immunosuppressive regimens (Ronco et al., 2021).

Beyond inducing short-term remission, TWG therapy was also associated with a significantly reduced risk of relapse (RR = 0.56; 95% CI: 0.37–0.86; p = 0.008), supporting its potential to achieve sustained long-term remission. No significant difference was observed in the overall incidence of adverse events (RR = 0.93; 95% CI: 0.65–1.34). Although a non-significant trend toward increased gastrointestinal side effects was noted in a subset of studies, the overall safety profile from the reported data suggests that many adverse reactions may be manageable in a clinical setting. However, this conclusion is tempered by the inconsistent reporting of adverse events across studies, as discussed in the limitations. TWG also demonstrated potential as a steroid-sparing agent, which could help reduce cumulative toxicity associated with long-term glucocorticoid use.

Regarding treatment strategy, this analysis indicates that TWG combined with low-to-medium-dose corticosteroids is a particularly promising regimen for maintaining remission. This combination can synergistically enhance immunomodulatory effects, improving efficacy over monotherapy while reducing long-term steroid-related toxicity through a “steroid-sparing” effect (Bi et al., 2024). Although combination therapy with calcineurin inhibitors (e.g., tacrolimus) has also proven effective, the optimal regimen should be individualized based on patient risk profiles and tolerance.

For clinical practice, the evidence summarized in this review suggests that TWG could be considered a complementary or alternative option for IMN management, particularly in regions where it is already in use. However, close monitoring for potential adverse effects, such as hepatotoxicity, is essential. Future research should prioritize head-to-head comparisons between TWG and current standard therapies (e.g., rituximab), optimize dosing regimens, and explore biomarkers for predicting treatment response to advance the development of personalized therapeutic strategies.

This review has several limitations. First, and most importantly, the interpretation of our findings is substantially limited by the methodological quality of the included evidence. Seventeen of the twenty (85%) studies were judged to have a high risk of bias, primarily due to inadequacies in randomization, allocation concealment, and blinding. It is well-established that such methodological weaknesses systematically lead to an overestimation of treatment effects in meta-analyses. Consequently, the magnitude of the beneficial effects observed for TWG, particularly for total clinical response and complete remission, should be interpreted with great caution. Second, adverse events were inconsistently and likely incompletely reported across studies. This fundamental limitation means that our finding of no significant difference in overall adverse event rates (RR = 0.93) should not be misinterpreted as evidence of safety, as it probably fails to capture the full spectrum and incidence of known toxicities such as hepatotoxicity, nephrotoxicity, and reproductive toxicity associated with TWG use (Wei et al., 2019). Third, the exceptionally high heterogeneity (I^2^ > 95% for key outcomes such as urinary protein and serum albumin) underscores the substantial clinical and methodological diversity among the included studies. While we performed prespecified subgroup analyses, they failed to fully elucidate the sources of this heterogeneity, which likely stems from unmeasured factors such as variations in baseline patient characteristics, precise TWG dosing, and adjunctive treatments. This inherent variability cautions against the over-interpretation of the pooled effect estimates. Fourth, several studies lacked key baseline data, including mean age and disease staging, which may have introduced additional heterogeneity and limited the precision of subgroup analyses. These omissions were reflected in the risk of bias assessments. Fifth, only a few studies included long-term follow-up, restricting conclusions about the durability of clinical remission. Sixth, although Egger’s test did not indicate publication bias, the absence of trial registry data and unpublished results means such bias cannot be fully ruled out. Finally, a critical limitation is the exclusive inclusion of studies from China. This geographic and ethnic homogeneity severely restricts the generalizability of our findings. Genetic factors, such as variations in HLA-DQA1 and PLA2R1 alleles, which differ across populations and are linked to IMN susceptibility and progression (Stanescu et al., 2011), may significantly influence treatment response to TWG. Therefore, the efficacy and safety observed here may not be directly applicable to non-Chinese populations, highlighting an urgent need for international, multi-ethnic studies.

Despite its therapeutic benefits, the potential toxicities of TWG cannot be overlooked. Our analysis, consistent with the known safety profile, indicates that the most common adverse effects include hepatotoxicity (elevated liver enzymes), gastrointestinal disturbances (nausea, diarrhea), and reproductive toxicity (menstrual irregularities in women, potential effects on male fertility). It is imperative that clinicians employing TWG maintain a high index of vigilance, implementing regular monitoring of liver function and providing appropriate patient counseling, particularly to those of reproductive age. The risk-benefit ratio should be carefully evaluated for each individual patient.

## 5 Conclusion

In conclusion, TWG represents a promising yet incompletely evaluated therapeutic option for IMN. The summarized evidence supports its potential use, especially in settings where it is already established, but does not yet justify its universal application. The translation of these findings into global clinical practice hinges on the completion of international, comparative-effectiveness trials. In the interim, if TWG is considered, its employment must be accompanied by a thorough risk-benefit discussion and a structured monitoring protocol for its characteristic toxicities.

## Data Availability

The original contributions presented in the study are included in the article/Supplementary Material, further inquiries can be directed to the corresponding author.

## Abbreviations

CI, Confidence Interval; KDIGO, Kidney Disease: Improving Global Outcomes; DQA1, DQ Alpha Chain 1; ESRD, End-Stage Renal Disease; IMN, Idiopathic Membranous Nephropathy; RAGE, Receptor for Advanced Glycation End products; CNI, Calcineurin Inhibitors; HLA, Human Leukocyte Antigen; HKSJ, Hartung–Knapp–Sidik–Jonkman Adjustment; IMN, Idiopathic membranous nephropathy; I^2^, I-squared; AGEs, Advanced Glycation End products; IPD, Individual patient data; MN, membranous nephropathy; NOS, Newcastle-Ottawa Scale; THSD7A, Thrombospondin type-1 domain-containing 7A; SEMA3B, Semaphorin 3B; PLA2R1, Phospholipase A2 Receptor 1; PRISMA, Preferred Reporting Items for Systematic Reviews and Meta-Analyses; PICOS, Population-Intervention-Comparison-Outcome-Study design; RR, Risk ratios; RCT, Randomized controlled trial; TNF-α, Tumor Necrosis Factor-α; SMD, Standardized Mean Difference; TWG, Tripterygium wilfordii glycosides; CNKI, China National Knowledge Infrastructure.
