# Supplementary material for: Clinical efficacy and safety of tripterygium wilfordii glycosides in the treatment of idiopathic membranous nephropathy: a systematic review and meta-analysis
Source: Front Pharmacol. 2025 Oct 31;16:1652789. doi: 10.3389/fphar.2025.1652789 (PMC12615218; doi:10.3389/fphar.2025.1652789)
Supplement: Supplementary file 1 [file Supplementaryfile1.doc]

Supplementary Material Table S1:

*Database search strategies*

| NO. | Database | Date  Searched | Search Strategy (Keywords/Subject Headings) |
| --- | --- | --- | --- |
| 1 | PubMed (NCBI) | 11.09.2025 | ("tripterygium"[MeSH] OR "tripterygium wilfordii" OR "tripterygium glycosides" OR "TWG") AND ("glomerulonephritis, membranous"[MeSH] OR "membranous nephropathy" OR "idiopathic membranous nephropathy" OR "IMN") |
| 2 | Embase (Ovid) | 11.09.2025 | ('tripterygium wilfordii'/exp OR "tripterygium glycosides" OR "TWG") AND ('membranous nephropathy'/exp OR "idiopathic membranous nephropathy" OR "IMN") |
| 3 | CENTRAL (Cochrane Library) | 11.09.2025 | ("tripterygium wilfordii" OR "tripterygium glycosides" OR "TWG"): ti, ab, kw AND ("membranous nephropathy" OR "idiopathic membranous nephropathy" OR "IMN"): ti, ab, kw |
| 4 | Web of Science Core Collection | 11.09.2025 | TS=("tripterygium wilfordii" OR "tripterygium glycosides" OR "TWG") AND TS=("membranous nephropathy" OR "idiopathic membranous nephropathy" OR "IMN") |
| 5 | CNKI | 11.09.2025 | SU=("tripterygium wilfordii" OR "tripterygium glycosides" OR "TWG") AND SU=("membranous nephropathy" OR "idiopathic membranous nephropathy" OR "IMN") |
| 6 | Wanfang Data | 11.09.2025 | Topic=("tripterygium wilfordii" OR "tripterygium glycosides" OR "TWG") AND Topic=("membranous nephropathy" OR "idiopathic membranous nephropathy" OR "IMN") |
| 7 | VIP | 11.09.2025 | R=("tripterygium wilfordii" OR "tripterygium glycosides" OR "TWG") AND R=("membranous nephropathy" OR "idiopathic membranous nephropathy" OR "IMN") |

Table S2. Representative Examples of Studies Excluded During Full-Text Review and Reasons for Exclusion

| No. | Study ID (Author, Year) | Primary Reason for Exclusion | Conference |
| --- | --- | --- | --- |
| 1 | Ai et al. (2022) | Control group received TWG (violates comparator criteria). | Eiliman, Wang Shun. A study on the efficacy of combined Tripterygium wilfordii glycosides and low-dose corticosteroids in the treatment of idiopathic membranous nephropathy. Shaanxi Journal of Traditional Chinese Medicine, 2022, 43(06): 712-714.  DOI: CNKI:SUN:SXZY.0.2022-06-008. |
| 2 | Chen et al. (2022) | Control group received Tacrolimus (an intervention component), not pure conventional therapy. | Chen Yuling, Shen Peng, Sun Dandan. Therapeutic effects of low-dose tacrolimus combined with Tripterygium wilfordii glycosides in adult idiopathic membranous nephropathy. Medical Theory and Practice, 2022, 35(03): 439-441.  DOI: 10.19381/j.issn.1001-7585.2022.03.032. |
| 3 | Feng et al. (2023) | Control group used Jin Shui Bao (herbal medicine), not conventional therapy. | Feng Si, Hu Yuan, Xu Shizhang, et al. Efficacy of low-dose corticosteroids combined with Tripterygium wilfordii in the treatment of idiopathic membranous nephropathy in middle-aged and elderly patients. Modern Diagnosis & Treatment, 2023, 34(10): 1480-1482.  DOI: CNKI:SUN:XDZD.0.2023-10-021. |
| 4 | Gao et al. (2023) | Control group included TWG (violates comparator criteria). | Gao Yu, Wu Fuxian, Liu Yangmei. Clinical analysis of Tripterygium wilfordii glycosides, leflunomide, and low-dose corticosteroids in the treatment of idiopathic membranous nephropathy. Chinese and Foreign Medical Journal, 2023, 42(26): 90-93.  DOI: 10.16662/j.cnki.1674-0742.2023.26.090. |
| 5 | Guo et al. (2019) | Control group received TWG, not conventional therapy. | Guo Bohui, Li Yi, Li Xiangdong. Effects of total Rehmannia leaf glycosides combined with Tripterygium wilfordii glycosides on treatment efficacy and prognosis in membranous nephropathy patients. Journal of Clinical Nephrology, 2019, 19(01): 54-57+69.  DOI: CNKI:SUN:LCSB.0.2019-01-011. |
| 6 | Liu et al. (2020) | No control group receiving conventional therapy. | Liu Min, Zhuang Yongze. Relationship between treatment efficacy of hormone combined with Tripterygium wilfordii glycosides and serum anti-PLA2R antibody levels in adult idiopathic membranous nephropathy. Chinese Journal of Integrated Traditional Chinese and Western Medicine Nephrology, 2020, 21(02): 113-116.  DOI: CNKI:SUN:JXSB.0.2020-02-008. |
| 7 | Wang et al. (2016) | Control group received TWG, not conventional therapy. | Wang Changsheng. Clinical study on the combined use of Bu Yang Huan Wu decoction and Tripterygium glycosides in the treatment of idiopathic membranous nephropathy. Shandong University of Traditional Chinese Medicine, 2016. |
| 8 | Wu et al. (2013) | Control group received TWG, not conventional therapy. | Wu Qiong, Du Xuan Yi, Ren Miaomiao. Efficacy observation of Shulodiet and Tripterygium glycosides in the treatment of moderate to high-risk idiopathic membranous nephropathy. Journal of Harbin Medical University, 2013, 47(02): 160-163.  DOI: CNKI:SUN:HYDX.0.2013-02-018. |
| 9 | Xue et al. (2019) | Control group received TWG, not conventional therapy. | Xue Piliang, Niu Wenying, Li Liqi, et al. A prospective controlled study on the combined use of Tripterygium wilfordii glycosides and low-dose corticosteroids in the treatment of idiopathic membranous nephropathy. World Journal of Traditional Chinese Medicine, 2019, 14(03): 666-669. |
| 10 | Yan et al. (2019) | Control group received TWG, not conventional therapy. | Yan Sishi, Xu Ji, Qiu Junfei, et al. Clinical efficacy of low-dose tacrolimus combined with Tripterygium wilfordii tablets in the treatment of idiopathic membranous nephropathy. Chinese Higher Medical Education, 2019, (01): 134-135. DOI: CNKI:SUN:ZOGU.0.2019-01-074. |
| 11 | Yu et al. (2023) | Control group received Huang Kui capsule (not conventional therapy). | Guanghui Yu. Clinical study on the combined therapy of Si-miao Si-jun decoction and Tripterygium wilfordii multiglucoside tablets for idiopathic membranous nephropathy [D]. Shandong University of Traditional Chinese Medicine, 2023.  DOI: 10.27282/d.cnki.gsdzu.2023.001409. |
| 12 | Chen et al. (2017) | Confounding intervention (TWG + corticosteroids vs. supportive care only). | Li Chen, Luxi Cai. Efficacy and feasibility analysis of Tripterygium wilfordii multiglucoside combined with low-dose corticosteroids in the treatment of idiopathic membranous nephropathy [J]. Chinese Journal of Maternal and Child Health Research, 2017, 28(S1): 349.  DOI: CNKI:SUN:SANE.0.2017-S1-387. |
| 13 | Gong et al. (2018) | Intervention group received triple immunosuppressive therapy. | Jiachuan Gong. Effects of cyclophosphamide, total glucosides of Tripterygium wilfordii, and mycophenolate mofetil triple therapy on clinical outcomes and immunoglobulin levels (IgA, IgM, IgG) in patients with membranous nephropathy [J]. Chinese Journal of Clinical Physicians, 2018, 46(06): 689-691. DOI: CNKI:SUN:ZLYS.0.2018-06-023. |
| 14 | Li et al. (2016) | Confounding intervention (TWG + complex Chinese medicine vs. conventional immunosuppression). | Xiang Li. Therapeutic efficacy of kidney-tonifying and blood stasis-eliminating traditional Chinese medicine combined with Tripterygium wilfordii glycosides in adult idiopathic membranous nephropathy: a clinical observation [D]. Zhejiang University of Traditional Chinese Medicine, 2016. DOI: 10.27465/d.cnki.gzzyc.2016.000029. |
| 15 | Xie et al. (2019) | Co-intervention of low molecular weight heparin in the intervention group. | Qianhong Xie. Clinical efficacy of Tripterygium wilfordii glycosides combined with corticosteroids and low molecular weight heparin in the treatment of idiopathic membranous nephropathy [J]. Journal of Rational Drug Use, 2019, 12(17): 55-56. DOI: 10.15887/j.cnki.13-1389/r.2019.17.033. |
| 16 | Zhang et al. (2017) | Confounding intervention (TWG + leflunomide + corticosteroids vs. TWG monotherapy). | Shengzhi Zhang. Efficacy of leflunomide combined with Tripterygium wilfordii glycosides and low-dose corticosteroids in treating idiopathic membranous nephropathy [J]. Northern Pharmacy, 2017, 14(02): 94. DOI: CNKI:SUN:BFYX.0.2017-02-081. |
| 17 | Feng et al. (2011) | Study population includes patients under 18 years of age. | Shaozun Feng, Ping Zhang, Ling Xie. Clinical observation of leflunomide combined with Tripterygium wilfordii glycosides and low-dose corticosteroids in the treatment of idiopathic membranous nephropathy [J]. Chinese Community Physician (Medical Professional), 2011, 13(17): 52. DOI: CNKI:SUN:ZGSQ.0.2011-17-051. |
| 18 | Zhang et al. (2024) | Diagnostic method for IMN not explicitly stated. | Shaojun Zhang, Qiaohua Su. Clinical efficacy of tacrolimus combined with Tripterygium wilfordii glycosides in the treatment of idiopathic membranous nephropathy [J]. Chinese and Foreign Medical Research, 2024, 3(27): 42-44. DOI: CNKI:SUN:WYZY.0.2024-27-014. |
| 19 | Tu et al. (2021) | Population not limited to IMN; includes mixed CKD types without confirmation of IMN. | Xiao Tu, Mengdie Yang, Yayu Li, et al. Comparative study on the efficacy and safety of Kunxian capsules versus Tripterygium wilfordii glycosides in the treatment of chronic kidney disease [J]. Journal of Zhejiang University of Traditional Chinese Medicine, 2021, 45(06): 582-587+602. DOI: 10.16466/j.issn1005-5509.2021.06.003. |
| 20 | Xiong et al. (2020) | Study population not confirmed as IMN. | Zhihui Xiong, Xinyan Huang. Clinical efficacy and safety of low-dose tacrolimus combined with Tripterygium wilfordii glycosides for targeted therapy of idiopathic membranous nephropathy [J]. Journal of Rational Drug Use, 2020, 13(25): 80-81. DOI: 10.15887/j.cnki.13-1389/r.2020.25.035. |
| 21 | Zhang et al. (2021) | Study population not confirmed as IMN. | Zhang Jingting. Clinical Study on the Combined Use of Tao Chen Membranous Nephropathy Decoction and Hormones with Lei Gong Teng in the Treatment of Membranous Nephropathy [D]. Liaoning University of Traditional Chinese Medicine, 2021. DOI: 10.27213/d.cnki.glnzc.2021.000534. |
| 22 | Zhou et al. (2011) | Study population includes patients under 18 years of age. | Zhou Jing, Wu Min, Zhang Wenyen. Clinical Observation of Tripterygium Wilfordii Multiglycoside Tablets Combined with Prednisone in the Treatment of Pediatric Primary Nephrotic Syndrome [J]. Chinese Journal of Integrated Traditional Chinese and Western Medicine Nephrology, 2011, 12(10): 909-910. |
| 23 | Deng et al. (2013) | Ineligible study design. | Deng Yueyi, Xie Zhiyong, Wang Lin, et al. Study on the Protective Effect of Triptolide on Podocytes in a Rat Model of Membranous Nephropathy [J]. Chinese Journal of Integrated Traditional Chinese and Western Medicine Nephrology, 2013, 14(10): 847-850. |
| 24 | Hu et al. (2014) | Insufficient outcome data (key efficacy endpoints not reported). | Hu Weifeng, Chen Hongyu, Wang Yongjun, et al. Clinical Efficacy of Tripterygium Wilfordii Multiglycoside Tablets in the Treatment of Idiopathic Membranous Nephropathy [J]. Chinese Journal of Integrated Traditional Chinese and Western Medicine Nephrology, 2014, 15(8): 714-716. |
| 25 | Liu et al. (2018) | Conference abstract only (insufficient detail for data extraction). | Liu Chunfeng, Li Xue, Zhang Hong, et al. Preliminary Report on Multicenter Clinical Study of Tripterygium Wilfordii Multiglycoside in the Treatment of Idiopathic Membranous Nephropathy [C]. Proceedings of the 2018 Academic Conference of the Chinese Medical Association Nephrology Branch. 2018. |
| 26 | Wei et al. (2012) | Intervention not clearly defined/dosed (TWG dosage regimen unclear). | Wei Yongquan. Efficacy Analysis of Combined Tripterygium Wilfordii Multiglycoside and Valsartan in the Treatment of Primary Membranous Nephropathy [J]. China Medical Guide, 2012, 14(10): 1720-1721. |
| 27 | Zhang et al. (2011) | Conference abstract only (insufficient detail for data extraction). | Zhang Hong, Zhao Xiaofang, Li Jing, et al. Clinical Observation of Combined Tripterygium Wilfordii Multiglycoside and Prednisone in the Treatment of Idiopathic Membranous Nephropathy [C]. Proceedings of the 2011 Academic Conference of the Chinese Medical Association Nephrology Branch. 2011. |
| 28 | Sun et al. (2008) | Non-randomized study design (retrospective cohort) with significant baseline imbalances. | Sun Lincheng, Liu Hong, Xu Jian, et al. Efficacy Observation of Combined Tripterygium Wilfordii Multiglycoside and Hormones in the Treatment of Idiopathic Membranous Nephropathy [J]. Chinese Modern Doctor, 2008, 46(23): 56-57. |
| 29 | Zhou et al. (2015) | Non-randomized study design (retrospective cohort) with significant baseline imbalances. | Zhou Zhenzhong. Clinical Efficacy of Low-Dose Prednisone Combined with Tripterygium Wilfordii Multiglycoside in Elderly Patients with Primary Nephrotic Syndrome [J]. Modern Diagnosis and Treatment, 2015, 26(21): 4860-4861. DOI: CNKI:SUN:XDZD.0.2015-21-042. |
| 30 | Yan et al. (2019) | Diagnostic method for IMN not explicitly stated. | Yan Hong, Liu Jie, Wang Lu, et al. Clinical Study on the Combined Use of Tripterygium Wilfordii Multiglycoside and Low-Dose Tacrolimus in the Treatment of Idiopathic Membranous Nephropathy [J]. Chinese Journal of Clinical Pharmacology, 2019, 35(15): 1625-1628. |

Note: This table provides 30 representative examples of the 121 studies excluded after full-text review. The most common reasons for exclusion were: inappropriate control group (e.g., control group received the intervention under investigation, or received non-conventional therapy), confounding interventions, ineligible study population, and insufficient outcome reporting. The complete list is available from the corresponding author upon reasonable request.

In the aforementioned references, J denotes journal articles, D signifies doctoral dissertations, and C represents conference papers.
